# Enhancing engagement and self-efficacy through AI-supported Chinese language instruction: a mixed-methods intervention study

**DOI:** 10.3389/fpsyg.2026.1913558

**Published:** 2026-07-29

**Authors:** Xiuwen Zhai

**Affiliations:** 1 School of Foreign Languages and Literature, Guangxi University, Nanning, Guangxi Province, China; 2 Asia-Pacific (Southeast Asia) Institute for Translation and Intercultural Studies, Nanning, Guangxi, China

**Keywords:** AI-supported instruction, Chinese language learning, engagement, self-determination theory, self-efficacy

## Abstract

This study examined the impact of AI-supported Chinese language instruction on engagement and self-efficacy among Malaysian university students. A quasi-experimental intervention with two instructional groups, an AI-supported teaching group and a traditional teaching group, was employed using an explanatory sequential mixed-methods design. The three-month intervention involved 100 undergraduate students. Measures of engagement and self-efficacy were collected before and after the intervention to examine changes within and between groups. Mixed-design ANOVA revealed significant Group × Time interaction effects for both engagement (partial *η*^2^ = 0.165) and self-efficacy (partial *η*^2^ = 0.116), indicating significantly greater improvements in the AI-supported group than in the traditional group. Results showed that students in the AI-supported group experienced significant gains in engagement and self-efficacy, whereas the traditional group showed little change. Following the quantitative phase, semi-structured interviews were conducted to explain the quantitative findings. Qualitative analyses helped explain these findings, indicating that AI-supported activities fostered active engagement, enhanced cognitive processing, promoted positive emotional experiences, and increased confidence in Chinese language knowledge and production. The study highlights the potential of AI tools to enhance motivation and psychological outcomes in language learning and provides practical recommendations for implementing AI in the classroom through effective teacher support and task design.

## 1 Introduction

Artificial Intelligence (AI) has been increasingly transforming language learning, as it extends the opportunities for learners to access language input, practice language skills, and receive support in language learning (Pack and Maloney, 2023). This support can be especially beneficial in Chinese as a foreign language teaching since the students may require repeated exposure, practice, and immediate feedback to gain confidence in comprehending and applying the language (Xia et al., 2024). In many classroom settings, however, students may still be largely dependent on teacher explanations and limited practice in the classroom, limiting their engagement and build-up of confidence (Low et al., 2025). The problem is particularly pertinent in multilingual higher education contexts, including those with students studying Chinese in a linguistically diverse environment in which they have varying degrees of prior exposure to Chinese (Peng et al., 2025), like Malaysia. Despite the increasing interest in the use of AI in language education, there is still a lack of empirical research to determine if AI-assisted classroom learning can significantly enhance students’ learning experiences and psychological wellbeing (Razak et al., 2025; Solhi et al., 2023). Hence, the study on AI-assisted Chinese language learning could not only enrich the research area of technology-assisted language learning but also benefit the Chinese language teaching practice in the classroom (Wu et al., 2024).

Although AI-supported language learning has attracted considerable scholarly attention, most existing studies have focused on English language learning, particularly writing support, vocabulary acquisition, speaking practice, and automated feedback. Comparatively little empirical research has examined AI-supported Chinese as a Foreign Language (CFL) instruction, despite the distinctive linguistic challenges associated with learning Chinese, such as character recognition, tonal pronunciation, and productive language use. Moreover, evidence from multilingual higher education contexts remains particularly limited. In Malaysia, students differ substantially in their linguistic backgrounds, prior exposure to Chinese, and opportunities to use Chinese beyond the classroom, all of which may influence their responses to AI-supported instruction. Therefore, investigating AI-supported Chinese language learning in this multilingual context can provide important empirical evidence for understanding how AI may support learners’ engagement and self-efficacy in CFL education and extend the current literature beyond the predominantly English-focused research. Unlike previous studies that have primarily focused on AI-supported English language learning, the present study contributes to the literature by examining AI-supported Chinese language instruction in a multilingual Malaysian university context, employing an explanatory sequential mixed-methods design, and interpreting the findings through the perspective of Self-Determination Theory.

In this study, the impact of AI-assisted Chinese language learning on the engagement and self-efficacy of Malaysian university students is explored. Engagement was chosen since it is a comprehensive indicator of students’ behaviors, thinking, and emotions when learning Chinese, and self-efficacy was chosen because it is an indicator of students’ confidence in understanding and producing Chinese. To meet these concerns, an explanatory sequential mixed-methods approach was used. The quantitative phase involved a three-month quasi-experimental intervention using two intact classes. In one class, instruction was supported by AI while the other class was instructed in the traditional classroom environment. Students were given engagement and self-efficacy questionnaires pre- and post-intervention. The qualitative phase was done to explain the quantitative findings by using semi-structured interviews to explore students’ perceptions of their learning experiences. This study had two research questions: To what extent does AI-supported teaching result in changes in students’ engagement and self-efficacy compared to traditional teaching; and how do students’ experiences in the two instructional conditions explain the quantitative effects?

## 2 Literature review

### 2.1 AI-supported Chinese language instruction

The application of AI in language teaching and learning has increasingly demonstrated its significance in recent years, offering the possibility of improving language teaching and learning (Sun et al., 2025). AI can provide learners with personalized practice activities, real-time feedback, interactive exercises, and increased autonomy in learning (Cabrera-Solano et al., 2026). Through these features, students can interact with the target language more actively, and get customized guidance that adjusts to their individual needs in learning (Borges et al., 2026). AI can provide supplementary opportunities for language exposure and learning for L2FL learners, such as listening, speaking, reading, and writing both inside and outside the classroom (Razak et al., 2025). Although some of these studies may bear potential value, most of the current research has focused on English language learning, vocabulary learning, feedback for writing or speaking practice (Peng et al., 2025). There is a lack of empirical studies specifically focusing on AI-assisted Chinese language teaching, especially in a multilingual environment like Malaysia (Low et al., 2025). This lack of research indicates the necessity of empirical studies to assess the effects of integrating AI into teaching on student outcomes (Wang et al., 2025). The present study aims to fill this gap by performing a quasi-experimental intervention based on the comparison of AI-supported teaching strategy with traditional teaching strategy to determine the effects on engagement and self-efficacy (Wang et al., 2025).

AI-assisted teaching in Chinese language classrooms can offer structured opportunities for input, practice, feedback and communicative use (Gao et al., 2026). The incorporation of AI tools enables instructors to provide students with comprehensive language input and also to provide individualized exercises based on each student’s learning level (Wen et al., 2025). The instantaneous feedback enables learners to recognize and correct their errors immediately, which can enhance the accuracy of language expression and promote correct forms (Wang et al., 2026). Furthermore, AI applications can mimic conversations, role-play, and real-life situations, giving students an opportunity to practice Chinese in practical situations without the pressure of real communication (Zhou, 2026). Such learning features can foster a positive learning atmosphere, where students are more inclined to actively learn, cognitively process difficult materials, and feel positive emotions when learning a language (Zhang et al., 2025). The regular practice and scaffolding provided by AI-assisted instruction can also boost students’ self-confidence in their language skills and help them complete complex language exercises independently (Sun et al., 2026a). These benefits lay the groundwork to consider how AI-enhanced teaching could impact student engagement and self-efficacy (Wu et al., 2026).

### 2.2 Student engagement and student self-efficacy

The quality of student engagement is considered to be a core measure of learning quality in language learning and teaching (Macfarlane and Tomlinson, 2017). It is indicative of the level of engagement in class, mental effort in tasks and emotional dispositions toward learning (Zepke and Leach, 2010). Engagement is a multifaceted phenomenon in language learning, encompassing emotional, cognitive, and behavioral aspects (Hiver et al., 2024). Behavioral engagement is defined as students’ involvement, perseverance, and effort to finish the learning task; cognitive engagement includes learners’ use of strategies, deep processing, and willingness to understand language content; and emotional engagement relates to students’ interest, enjoyment, and attitudes toward learning (Wu et al., 2026). These three dimensions are of greater significance in the field of Chinese language education due to the fact that students must show active language processing, emotional motivation to communicate, and prolonged interaction with the target language, which is relatively difficult to master (Tong et al., 2024). Therefore, engagement could be a beneficial lens to examine whether a teaching approach is able to foster a more active and meaningful participation in the Chinese language classroom (Fan and Tian, 2022).

Student self-efficacy refers to learners’ beliefs about their ability to complete learning tasks and use the target language (Van Dinther et al., 2011). Self-efficacy influences language learning by determining students’ approach to difficulties, their amount of effort, and their perseverance in the face of obstacles (Sun et al., 2026b). Students who have high self-efficacy are more likely to undertake difficult tasks, make mistakes, and believe that they are improving in their learning (Al-Abyadh and Abdel Azeem, 2022). In Chinese language learning, self-efficacy has a particular significance in Chinese language learning as Chinese language learners have difficulties with pronunciation, characters, grammar, and communicative expression (Henderson et al., 2012). It can be interpreted from the receptive and productive language aspects (Chen and Yeung, 2015). Receptive self-efficacy is the students’ confidence in comprehending Chinese input (listening and reading texts) while productive self-efficacy is their confidence in Chinese output (speaking and writing; Hong et al., 2017). These dimensions collectively represent the students’ perception of their competence in comprehending and using Chinese effectively (Al-Abyadh and Abdel Azeem, 2022). Thus, to assess the effectiveness of Chinese language teaching and learning, the psychological outcome such as self-efficacy is important.

There is a strong relationship between student engagement and self-efficacy and the quality of student support. AI in Chinese language classrooms can potentially provide more opportunities for active student engagement, repetition, instant feedback, and personalized support for learning (Tong et al., 2024). Such features can stimulate students behavioral, cognitive, and emotional reactions, such as increased interest, fewer frustrations, and more learning tasks (Al-Abyadh and Abdel Azeem, 2022). Meanwhile, AI-assisted instruction can boost students’ self-confidence by letting them practice and feedback, experiencing improvement and growth (Zhou, 2026). Clear feedback and provision of opportunities for students to apply Chinese in low-pressure situations may promote their confidence in understanding and speaking Chinese (Wen et al., 2025). Hence, the study on engagement and self-efficacy can be studied together to gain a comprehensive view of the impact of AI-supported instruction on students’ learning experiences (Henderson et al., 2012). This link also prepares the ground for the interpretation of the AI-supported teaching in the light of self-determination theory.

Although engagement and self-efficacy are closely related in language learning, the present study conceptualizes them as two complementary motivational outcomes rather than assuming a direct causal relationship between them. Engagement reflects learners’ active behavioral, cognitive, and emotional involvement in learning activities, whereas self-efficacy represents learners’ confidence in their ability to successfully understand and use the target language. Drawing on Self-Determination Theory (SDT), AI-supported instruction is expected to facilitate both outcomes by creating learning environments that provide greater opportunities for autonomous participation, immediate feedback, repeated practice, and successful learning experiences. Because the present study was not designed to examine causal pathways between engagement and self-efficacy, these constructs are interpreted as parallel outcomes through which the motivational benefits of AI-supported Chinese language instruction can be understood.

### 2.3 Theoretical framework: self-determination theory

Self-determination theory is a helpful framework for understanding the impact of learning environments on students’ motivation and learning outcomes (Noels et al., 2000). This theory suggests that when students’ basic psychological needs are met, they are better positioned to become actively and sustainably engaged (Alamer et al., 2025). These are autonomy, competence, and relatedness (Al-Hoorie et al., 2025). Autonomy involves students’ experience of choice, initiative and ownership of learning. Competence is a sense of students that they can perform successfully through learning activities and improve by trying. Relatedness is based on the sense of students’ connection with teachers, peers and learning environment. In the field of language education, these three needs are of special significance, for students should be active in communication, should struggle with language problems, and should feel confident in using the target language. If classroom instruction caters for such requirements, students are more inclined to put in effort, feel good about their learning and have confidence in the progress of their learning.

In this study, AI-assisted Chinese language teaching is defined as a learning environment that can meet students’ underlying psychological needs. AI tools can provide customized learning materials, adaptable practice activities, and learning control in terms of pacing, thereby increasing autonomy. They can be used to support competence through repeated practice, immediate feedback and evidence of Chinese learning. They can also enhance relatedness through teachers using AI tools for interactive classroom activities, peer communication, and meaningful language use. AI-assisted teaching could promote students’ active involvement, deep understanding of language, and positive emotional state in the classroom through these need supports. It can also give a boost to students’ confidence in understanding and creating Chinese, so that students can experience successful task completion. Thus, SDT provides a logical model to explain how the implementation of AI-assisted learning could be beneficial to increase engagement and self-efficacy compared to traditional instruction.

## 3 Methodology

### 3.1 Research design

The study employed an explanatory sequential mixed methods design to examine the impact of AI-assisted language teaching on students’ engagement and self-efficacy in Chinese language learning in Malaysia. The quantitative phase was a quasi-experimental intervention of two instructional conditions: AI supported teaching group and traditional teaching group. In order to observe the changes in students’ engagement and self-efficacy within each condition, and the differences between these conditions, pre- and post-tests were administered. After the quantitative phase, the qualitative phase was carried out in order to explain and interpret the results, and to gain students’ learning experiences, perceived classroom participation and confidence in using Chinese. Semi-structured interviews with a subsample of both groups were conducted in this stage to gain understanding of the underlying reasons for why AI-supported instruction might have had a different impact on engagement and self-efficacy. Together, the mixed-methods design addressed the following research questions:

RQ1. To what extent does AI-supported teaching lead to changes in Malaysian students’ engagement and self-efficacy in Chinese language learning compared with traditional teaching?

RQ2. How do students’ experiences in AI-supported and traditional classrooms help explain the quantitative effects on engagement and self-efficacy?

### 3.2 Participants

This study was carried out in a public university in Malaysia. The subjects were undergraduate students who were studying Chinese as a foreign language and selected by convenience sampling. Two intact classes with the same language teacher were identified and assigned to two instructional conditions (AI-supported teaching group vs. traditional teaching group). There were 50 students in each group making a total of 100 students. There were 24 males and 26 females in the AI-supported teaching group, and 23 males and 27 females in the traditional teaching group. The age of the participants in the two groups varied from 18 to 20 years. A total of 20 students were selected after the quantitative phase for semi-structured interviews, 10 from AI supported teaching group and 10 from the traditional teaching group. Interviewees were from different groups of instruction and included all genders and different levels of involvement in the classroom. The numbers 1 to 10 were those who belonged to the traditional teaching group, and the numbers 11 to 20 were those who belonged to the AI-supported teaching group. Because the study adopted a quasi-experimental design in a natural classroom setting, two administratively pre-existing intact classes were assigned to the AI-supported and traditional instructional conditions. Independent-samples t-tests confirmed that the two groups did not differ significantly in engagement or self-efficacy at the pretest, indicating comparable baseline conditions prior to the intervention.

### 3.3 Instruments

#### 3.3.1 Engagement

Engagement was measured by using the nine-item scale designed by Eerdemutu et al. (2024) to measure the behavioral, cognitive, and emotional type of engagement, with three items for each dimension. The items were contextualized for Chinese language learning and rated on a five-point Likert scale ranging from 1 = strongly disagree to 5 = strongly agree. A sample item is “I put effort into learning in my Chinese language class.” The scale demonstrated acceptable reliability at both time points. Cronbach’s alpha was 0.775 at the pretest and 0.739 at the posttest for the overall scale, while the coefficients for behavioral, cognitive, and emotional engagement were 0.916, 0.820, and 0.878 at the pretest and 0.783, 0.861, and 0.855 at the posttest, respectively.

#### 3.3.2 Self-efficacy

The scale developed by Kutuk et al. (2023) was used to measure self-efficacy. The scale comprised a total of 11 items measuring two dimensions: L2 reception self-efficacy (5 items), and L2 production self-efficacy (6 items). The wording of the items was adjusted to the context of Chinese language learning. An example statement is “I can say in Chinese what I think about current affairs or my future plans.” Each item was answered on a five-point Likert scale from 1 = strongly disagree to 5 = strongly agree. At both times of measurement, the internal consistency of the scale was satisfactory. The overall scale’s Cronbach’s alpha coefficient was 0.885 at the pretest and 0.835 at the posttest. The alpha coefficient scores for both reception and production self-efficacy were 0.908 and 0.831 at the pretest and 0.793 and 0.923 at the posttest, respectively.

#### 3.3.3 Interview protocol

The semi-structured interview protocol was created to explain and elaborate the quantitative results, and to examine learning experiences of the students in the two conditions of instruction. The protocol consisted of questions regarding students’ participation in the classroom, cognitive effort during the intervention, emotional reaction, confidence in learning and Chinese language learning change perceived after the intervention. The students in both groups were questioned about their involvement in the classroom activities, their approach to learning challenges and their confidence in learning Chinese and producing it after the class. The extra questions for students in the AI-supported teaching group aimed to gauge the impact of AI tools on their engagement, practice, feedback experiences, and confidence development. In the traditional teaching group, questions were directed at the students’ experiences of teacher-led instruction, classroom practice and feedback. Probing questions were asked to prompt more specific examples to make sure that students’ explanations were clear. The protocol was created to provide a comparison between the two groups’ experiences, and to determine potential explanations for differences in engagement and self-efficacy.

### 3.4 Intervention procedure and data collection

The intervention was conducted in regular Chinese language classes at a public Malaysian university, over a three-month period. The students in both the groups were given the engagement questionnaire and self-efficacy questionnaire as pretest using google form. The traditional teaching group continued to use the traditional teaching method; the teacher explained the points of language, set up the practice exercise, and gave feedback in the traditional way. The AI-assisted teaching group, on the other hand, was given instruction where teachers used AI tools to aid language input, practice, assessment, and communicative use of Chinese in their teaching.

To ensure consistency across the intervention, both groups were taught by the same instructor using identical curricular content, learning objectives, instructional schedule, and classroom hours throughout the three-month intervention. The AI-supported group integrated generative AI tools into regular classroom instruction to facilitate language input, pronunciation practice, dialogue simulation, sentence construction, writing revision, and immediate formative feedback through teacher-designed learning activities. In contrast, the traditional group received teacher-led instruction consisting of vocabulary explanation, grammar instruction, textbook-based practice, classroom discussion, and teacher feedback without the use of AI-supported learning tools. Prior to the intervention, the instructor prepared standardized lesson plans and became familiar with the pedagogical use of the AI tools to ensure consistent implementation across sessions. The intervention was conducted during regular weekly Chinese language classes, and comparable instructional time and learning tasks were maintained in both groups so that the primary difference between the two conditions was the integration of AI-supported instructional activities.

Students in both groups filled out the same questionnaires via Google Forms as a posttest after the intervention. The semi-structured interviews were completed 1 week post-test using Zoom. Interviews were conducted for about 30–50 min each and recorded on audio with the consent of the interviewees. Informed consent was obtained prior to data collection period and all participants were told the purpose of the study, that participation was voluntary, that it was confidential and they had the right to withdraw at any time.

### 3.5 Data analysis

The quantitative data analysis was conducted in several steps. Initial data was analyzed to determine the presence of missing values and distributional characteristics. Skewness and kurtosis were considered to determine the approximate normality of the data. Next, descriptive statistics were obtained for students’ pre- and post-test engagement scores and self-efficacy scores. Two-way mixed-design ANOVAs were run separately for engagement and self-efficacy to investigate intervention effects. Instructional group was designated as the between-subjects factor (traditional teaching group and the AI-supported teaching group), and time (pretest and posttest) was designated as the within-subjects factor. Simple effects analyses were conducted to determine differences between groups at each time point and changes within each group when significant group × time interaction effects were found.

Qualitative data was transcribed verbatim and analyzed in six steps as outlined by Braun and Clarke (2006) for thematic analysis. The analysis was designed to provide explanations and enhancement of the quantitative findings by comparing students’ learning experiences in the two instructional conditions. The transcripts were independently read by two researchers who produced initial codes, and patterns were identified for classroom participation, cognitive effort, emotional experience, comprehension confidence, production confidence and perceived progress. Then the codes were generated independently, and inter-coder agreement was greater than 90%. Any differences that arose were discussed, and consensus was reached. Following this process, related codes were organised into themes that highlighted differences between traditional and AI-supported groups. These themes were utilized to conceptualize the difference between the two groups on their engagement and self-efficacy outcomes.

## 4 Results

### 4.1 Quantitative results

#### 4.1.1 Descriptive statistics

Prior to the intervention, both groups indicated about the same engagement and self-efficacy level as displayed in Table 1. The mean scores of the two variables after the intervention indicated that the AI-supported teaching group obtained higher scores, while the traditional teaching group obtained scores with minimal changes. There were no significant deviations from normality, as indicated by the values of skewness and kurtosis, which were within acceptable limits (Kline, 2023).

**Table 1:** Descriptive statistics for engagement and self-efficacy by group and time.

Variable	Group	M	SD	Skewness	Kurtosis
Pre-engagement	AI-supported teaching	3.564	0.541	0.389	−0.182
	traditional teaching	3.500	0.416	−0.245	−0.508
Pre-self-efficacy	AI-supported teaching	3.289	0.653	0.169	−0.672
	traditional teaching	3.204	0.538	−0.066	0.007
Post-engagement	AI-supported teaching	4.053	0.337	0.544	0.325
	traditional teaching	3.544	0.413	−0.361	−0.730
Post-self-efficacy	AI-supported teaching	3.864	0.423	−1.041	0.811
	traditional teaching	3.285	0.587	0.286	−1.377

#### 4.1.2 Pre-intervention group comparability

Independent-samples t-tests were used to compare the two groups to see if they were comparable prior to the intervention. On the pretest engagement, there was no significant difference between the AI supported teaching group and the traditional teaching group, *t*(98) = 0.667, *p* = 0.506. The equal variance assumption was not met for pretest self-efficacy; the adjusted result is shown. The difference between the two groups was also not significant, *t*(94.574) = 0.714, *p* = 0.477. These findings showed that there was no difference between the two groups in engagement and self-efficacy prior to the intervention.

#### 4.1.3 Intervention effects

A summary of the mixed-design ANOVA results is presented in Table 2. A significant difference due to time and between instructional groups occurred for engagement. Further, a significant interaction between time and group indicated that the change from pretest to posttest was not the same for the two groups. In other words, the engagement development gamut was different between students who had been taught by AI-supported and traditional teaching. The same general pattern resulted for self-efficacy scores. The time and group factors varied significantly, as did the time x group interaction. This means that there was a shift in students’ self-efficacy when they received AI-assisted or conventional instruction. The values of 0.01 to less than 0.06 represent small effects, 0.06 to less than 0.14 represent medium effects, and 0.14 or higher represent large effects, based on Cohen’s (1988) guidelines for partial *η*^2^. A large interaction effect was found for engagement and a medium effect for self-efficacy.

**Table 2:** Results of two-way mixed-design ANOVAs for engagement and self-efficacy.

Variable	Source of variation	Type III sum of squares	*df*	Mean square	*F*	*p*	Partial *η^2^*
Engagement	Within-subjects (time)	3.556	1	3.556	27.889	***	0.222
	Time × Group	2.469	1	2.469	19.368	***	0.165
	Error (time)	12.494	98	0.127			
	Between-subjects (group)	4.109	1	4.109	16.580	***	0.145
	Error (between)	24.286	98	0.248			
Self-efficacy	Within-subjects (time)	5.385	1	5.385	22.841	***	0.189
	Time × Group	3.035	1	3.035	12.872	0.001	0.116
	Error (time)	23.105	98	0.236			
	Between-subjects (group)	5.505	1	5.505	14.343	***	0.128
	Error (between)	37.614	98	0.384			

***p < 0.001.

Tables 3, 4 give additional details of significant interaction effects. From the pretest to post-test, the AI supported teaching group had a significant increase of engagement while the traditional teaching group did not. Between-group comparisons indicated that there was no significant difference between the two groups before the intervention, and the scores of the AI supported teaching group were significantly higher than the traditional teaching group after the intervention. This was also the case for self-efficacy. The students in the AI-supported teaching group indicated a significant pretest–post-test difference and the students in the traditional teaching group indicated no significant difference as a function of time. The two groups did not differ at pretest and the AI group had significantly greater self-efficacy than the traditional teaching group at post-test.

**Table 3:** Simple effects analyses for engagement.

Comparison	I (time)	J (time)	Mean differences (I − J)	SE	*p*	95% CI	
						Lower	Upper
Within-subjects comparisons							
AI-supported teaching	Post	Pre	0.489	0.071	***	0.347	0.631
Traditional teaching	Post	Pre	0.044	0.071	0.535	−0.097	0.186
Between-subjects comparisons							
Pre	AI-supported teaching	Traditional teaching	0.064	0.097	0.506	−0.127	0.256
Post	AI-supported teaching	Traditional teaching	0.509	0.075	***	0.359	0.658

***p < 0.001.

**Table 4:** Simple effects analyses for self-efficacy.

Comparison	I (time)	J (time)	Mean differences (I − J)	SE	*p*	95% CI	
						Lower	Upper
Within-subjects comparisons							
AI-supported teaching	Post	Pre	0.575	0.097	***	0.382	0.767
Traditional teaching	Post	Pre	0.082	0.097	0.402	−0.111	0.275
Between-subjects comparisons							
Pre	AI-supported teaching	Traditional teaching	0.085	0.120	0.477	−0.152	0.323
Post	AI-supported teaching	Traditional teaching	0.578	0.102	***	0.375	0.781

***p < 0.001.

### 4.2 Qualitative results

#### 4.2.1 Students’ experiences of engagement in AI-supported and traditional classrooms

##### 4.2.1.1 Theme 1: from teacher-dependent participation to AI-supported active involvement

Behavioral engagement varied between the two groups with more teacher-dependent engagement in the traditional group and more active and sustained engagement in the AI-supported group. This gap indicates that in addition to the desire to learn Chinese, the type of tasks and the opportunities for interaction during Chinese teaching also influenced the students’ participation. There seemed to be fewer opportunities for students to act, react and revisit in the context of the teaching and learning process. Participant 5 exemplifies this behavior: participation is largely prompted by teacher questioning instead of the participant’s own actions. The student may also feel that traditional classroom participation is a public, infrequent and emotionally risky event because of anxiety about getting things wrong. Participant 13, on the other hand, demonstrates the use of AI-supported tasks to foster repeated responses, dialogue practice, and instant correction. All of these features appeared to make participation more continuous and less pressured and thereby made the student more willing to participate regularly. The AI-supported behavioral engagement and the teacher-dependent behavioral engagement are contrasted with the following excerpts.

Participant 5, traditional group: “*During most classes, I waited for the teacher to call on me before I spoke or completed tasks. I felt nervous about making mistakes in front of classmates, so I often stayed quiet or only did what was strictly required. Participation felt occasional rather than continuous, and I could not engage actively without prompts from the teacher.*”

Participant 13, AI-supported group: “*The AI exercises made me want to respond and practice continuously. I could answer questions, try dialogues, and get instant feedback, which let me correct mistakes immediately. This made me feel more involved and willing to participate in class every day, not just when the teacher asked me to speak.*”

##### 4.2.1.2 Theme 2: from surface task completion to deeper cognitive engagement

There were also some differences in the level of cognitive engagement in the two groups, with the traditional group students reporting more surface-level engagement in the tasks and the AI group students indicating a higher level of reflection on the use of language. This difference implies that the feedback and learning supports provided in the classroom activities affected the Chinese language content processing of students. Traditional instruction involved explanations from the teacher and doing exercises, but with less immediate opportunities for comparing, revising and thinking over answers. This is evidenced by participant 7 who states that learning is mostly listening, memorising and waiting for the teacher’s correction. This means there was cognitive effort, but it was not self-directed and it was not immediately associated with reflection. By contrast, Participant 16 notes that AI feedback enabled them to identify mistakes, to compare other possible expressions and to rewrite answers multiple times. These activities made the student more aware of words, grammar and sentence meaning. The following excerpts illustrate how AI-assisted learning fostered more profound cognitive processing than more mundane task completion.

Participant 7, traditional group: “*In the traditional class, I mainly listened to the teacher, made notes of key points, and when doing exercises, waited for the teacher to check the answer and tried to remember grammar rules, but I failed to think thoroughly about the principle of why the answer was wrong and how to improve it.”*

Participant 16, AI supported group: *“The AI-supported tool did not just indicate if I got an answer wrong, it also provided me with an alternative way to formulate the sentence and the explanation. I compared my answer with their suggestion, adjusted some of the content and tried again, which made me think about Chinese grammar and meaning more deeply*.”

##### 4.2.1.3 Theme 3: from anxiety and hesitation to more positive emotional engagement

Another difference between the two groups was in emotional engagement, with the traditional-group students expressing more anxiety and hesitation, and the AI-supported students expressing more interest, comfort, and enjoyment in learning Chinese. This difference suggests that emotional engagement was highly correlated to the students’ perception of safe and motivating classroom. In traditional classroom settings, speaking or answering questions would often be equated with being evaluated by the public and thus traditional students may not be as willing to speak or answer questions as they would be emotionally. Participant 2 echoes this experience in connecting Chinese learning to nervousness, fear of making mistakes, and hesitation to speak in front of the classmates. This indicates that emotional obstacles may have affected the students’ persistence in Chinese learning, though they know the necessity of Chinese learning. In contrast, Participant 18 finds the AI-supported activities more enjoyable and less stressful as they provided opportunities for trial, correction, and repeated practice before sharing with others. This lowered pressure seemed to introduce positive feelings toward Chinese learning. The following excerpts show the differences in emotional involvement in the two instructional conditions.

Participant 2, traditional group: “*I knew the importance of Chinese, but I found myself sometimes a little nervous in class, when the teacher asked me question, I was worried about my pronunciation and grammar would not be correct, and other students might hear me, so sometimes I did not enjoy speaking activities, although I wanted to do more.”*

Participant 18, AI-supported group: *“The AI activities have helped to make Chinese class more interesting and less stressful for me. I could get feedback on my answer and repeat it if I needed to, before posting for others to see. I felt secure — because my mistakes were not so embarrassing. Gradually I got more and more interested in the activities and happier in using Chinese in class.*”

#### 4.2.2 Students’ experiences of self-efficacy in AI-supported and traditional classrooms

##### 4.2.2.1 Theme 4: from uncertain comprehension to stronger confidence in understanding Chinese input

There were differences in the self-efficacy of understanding Chinese input between the two groups, with traditional group students demonstrating lower confidence and AI supported students indicating higher confidence in understanding Chinese input. The difference indicates that the scaffolding and feedback provided in the Chinese learning process had an impact on students’ learning confidence. In traditional classes, students relied heavily on teacher explanation and class content, and sometimes had difficulty understanding the content, or could not ask questions right away. Participant 4 exemplifies this by saying that he is not sure when hearing Chinese or reading new sentences without immediate assistance. This means that lack of personalized support might help to reduce students’ confidence in the comprehension of Chinese input. Participant 14, on the other hand, reports that AI tools were useful in understanding the meaning of the words, sentences, and pronunciation rapidly. These types of support seemed to create comprehension more manageable and to help the student feel more in control of input processing. Here are some examples of this contrast in receptive self-efficacy.

Participant 4, traditional group: “*When I listened to Chinese sentences in class, I sometimes understood only part of the meaning. If the teacher moved to the next activity, I did not always ask again because I felt shy. When reading, unfamiliar words also made me unsure. I could finish the task, but I was not confident that I really understood everything.*”

Participant 14, AI-supported group: “*The AI-supported tool helped me to understand Chinese input more clearly, when I did not understand a word or sentence, I could check its meaning, pronunciation, and example immediately, which made listening and reading less confusing and easier to understand in a self-study way, and when facing new Chinese materials, I could feel more confident*.”

##### 4.2.2.2 Theme 5: from limited expression to greater confidence in producing Chinese output

Moreover, there were differences among students’ confidence in Chinese output: traditional group students indicated that they had low self-confidence in Chinese speaking and writing, while AI supported students indicated that they had high self-confidence in Chinese output. This contrast suggests that opportunities for language production preparation, revision, and practice influenced output confidence. Traditional classes would require students to give responses to questions in front of others and given little time for correction, students were wary of speaking or writing. This is echoed by Participant 8 who expressed worries regarding pronunciation, grammar and accuracy of sentences which limited their inclination to communicate ideas in Chinese. This indicates that productive self-efficacy was limited by the fear of error or inadequate production. In contrast, Participant 17 demonstrates the use of AI to support learning as a safe environment to write and rewrite sentences, practice expressions, and rehearse speech before sharing in class. These opportunities enabled the student to be more successful in language production. The following excerpts show how AI assisted practice helped to enhance students’ confidence in speaking and writing Chinese.

Participant 8, traditional group: “*When I had to speak Chinese in class, I was not very confident, I was afraid of the pronunciation and used to give short answers, and in writing, I also relied on the examples in the textbook, I wanted to write more, but I was afraid of making my sentences sound strange*.”

Participant 17, AI supported group: “*When using the AI activities, I could write my sentence and then see how to improve it. I could also get some practice speaking before speaking to classmates. My answer got better after a few tries and I got more self-assured. I wasn’t just copying examples anymore. I began to think that I could create Chinese on my own*.”

##### 4.2.2.3 Theme 6: from unclear progress to feedback-driven confidence building

The third difference was related to the way students noticed their progress, where traditional group students indicated less visible evidence of progress and AI supported students indicated that they had increased confidence with repeated feedback. Findings indicate that self-efficacy was not only associated with performance at the end of the course, but also to the detection of incremental improvement in learning. The traditional group typically received feedback only during a few timeframes in class, and students were at time not certain that they were getting better. Participant 10 does so by describing that there was a lack of progress as errors were only corrected at the end of the activities. This took a while to make confidence soar. Participant 20, however, characterizes the AI feedback as “immediate, repeatable [which] can be seen as the errors go away across the tries.” This seen improvement contributed to practice as proof of capability. The following extracts illustrate the different types of feedback and their impact on students’ confidence about their Chinese learning progress.

Participant 10, traditional group: “*Sometimes I was not sure if my Chinese was improving. The teacher corrected our work, but usually after we finished the activity or after class. I could see some mistakes, but I did not always know how much better I became. Because the progress was not very clear, my confidence changed only a little.*”

Participant 20, AI-supported group: “*The most helpful part was seeing my progress immediately. At first, my answer had many problems, but after checking the AI feedback and trying again, I could see fewer mistakes. This made me feel that practice really worked. When I noticed my own improvement, I became more confident about learning Chinese.*”

## 5 Discussion

### 5.1 Effects of AI-supported instruction on engagement and self-efficacy

Results from the quantitative analysis showed that there were significant differences between the two instructional groups for engagement and self-efficacy. The results revealed that students in the AI-supported teaching group exhibited a pretest to posttest increase in engagement and self-efficacy, while students in the traditional teaching group did not exhibit significant changes in either. The mixed-design ANOVA revealed a significant group × time interaction for both variables, which confirmed that the instructional condition was a critical factor in determining students’ outcomes. Effect sizes for engagement and self-efficacy were medium to large, suggesting the practical significance of these differences. These findings suggest that AI-assisted teaching could offer valuable improvements for students’ learning in Chinese classrooms. Results give a quantitative base to understand the potentiality of AI tools for influencing students’ self-confidence in using Chinese language and the students’ active engagement in learning tasks. Each of these variables is now described in detail, with comparisons to previous studies to put these findings into perspective.

The AI-supported group showed significant gains in students’ self-efficacy in both receptive and productive language skills. Students indicated that their Chinese ability to understand speech and written language is improved, and their confidence in speaking and writing is improved. The traditional group, in comparison, demonstrated little growth, indicating that the traditional teacher-led instruction model did not offer enough opportunities for learners to develop confidence. These findings align with previous studies which found that individualized feedback, repeated practice and scaffolding of tasks can improve language learners’ self-efficacy (Al-Abyadh and Abdel Azeem, 2022; Kutuk et al., 2023). The present findings, however, go beyond the findings of the majority of the previous studies in English contexts to Chinese as a foreign language in a multilingual Malaysian university setting. These enhancements indicate that AI-driven interventions can help in creating mastery experiences, minimizing performance anxiety, and providing instant feedback, which all lead to improved self-efficacy. The findings present further evidence of the need for instructional design for the explicit support of learners’ confidence in comprehension and production as a result of technology enhanced teaching.

Terms of engagement, the AI-supported group saw a significant increase in all behavioral, cognitive, and emotional aspects while the traditional group has not changed much. Students’ behavioral involvement grew as they joined in more class activities, simulated dialogues, and completed tasks. Cognitive engagement was enhanced by deeper processing, strategy use, and reflection, all with the help of AI feedback. Emotional involvement was enhanced by increasing students’ interest and decreasing their anxiety and positive attitudes toward Chinese learning. These results resonate with previous studies that have shown that interactive and personalized learning environments can lead to engagement in foreign language classrooms (Eerdemutu et al., 2024; Zhou, 2026). Significantly, this research applies these findings to Chinese teaching with the aid of AI, demonstrating that AI can promote continuous engagement and emotional connection to the Chinese language, even for a difficult foreign language. The engagement results found in this study may offer a possible explanation for the self-efficacy results as both factors appear to go hand in hand. Qualitative results will be discussed in the next section to understand how students’ experiences in AI-supported classroom and traditional classroom influenced these changes.

### 5.2 Students’ perceptions underlying engagement and self-efficacy changes

The qualitative analysis brought out the quantitative results by illustrating students’ experience of AI-supported and traditional classrooms in a nuanced way. In general, students in the AI-assisted group reported higher levels of engagement, greater thoughtfulness, more positive feelings, and greater self-confidence in learning and creation in Chinese. The traditional students, in contrast, were more likely to say that they were more involved with the teacher, had less chance to rewrite, and found it less obvious that they were making any progress. The patterns indicate that engagement and self-efficacy may be influenced by the learning environment, and not only by students’ willingness. Sustaining student engagement and confidence stems from the repeated opportunities to act, get feedback, improve and enjoy getting better that happens through AI-supported activities. Traditional instruction was structured and teacher directed, but provided less opportunity for practice and individualized assistance. Therefore, the qualitative results contribute to clarifying the quantitative results of higher engagement and self-efficacy in the AI-supported group.

The students’ experiences focused on engagement and revealed differences in the behavioral, cognitive and emotional components. AI-assisted classrooms seemed to promote active engagement, in-depth understanding, and emotional commitment through interactive learning, dialogue simulations, and iterative exercises. In comparison, students in traditional classrooms frequently reported being engaged primarily by answering teacher questions, doing not much thinking, and feeling somewhat comfortable and somewhat interested. This finding is consistent with previous research which found that technology-assisted language learning can increase learners’ engagement through opportunities for action, reflection and collaborative interaction (Al-Abyadh and Abdel Azeem, 2022; Tong et al., 2024). The qualitative evidence supports the quantitative evidence by providing insight into why the AI-supported group was seen to have significant increases in engagement and the traditional group did not. All in all, the results highlight the need for task design, immediacy of practice and interactive feedback in encouraging sustained engagement in second language classrooms.

In terms of self-efficacy, the students’ perception indicated that AI-assisted instruction boosted comprehension and production self-efficacy. Through the repetition, instant feedback and introspection in the AI activities, students felt their improvement and became more confident in understanding and using Chinese. But traditional classroom participants indicated that they felt that their confidence in using Chinese to communicate was limited, especially in speaking and writing, because of their dependence on teacher feedback and lack of opportunities to practice. The results of this study align with previous studies on the importance of experiences of success, timely corrective feedback, and opportunities to self-monitor in the development of self-efficacy in language learning (Chen and Yeung, 2015; Henderson et al., 2012). Qualitative data was used to further describe the quantitative data, by focusing on the ways in which the instructional conditions impacted students’ perceived competence. In AI-assisted learning, students had opportunities for repeated success, leading to a gradual improvement in their self-perceptions. This indicates that the large differences in self-efficacy scores among the AI group was not just a statistical finding, but one where students’ confidence had also improved, likely impacting future engagement and learning behaviors.

### 5.3 Theoretical and practical implications

Although the present findings can be interpreted through the lens of Self-Determination Theory (SDT), the study did not directly measure learners’ psychological needs for autonomy, competence, and relatedness. Therefore, SDT is adopted as an interpretive framework rather than a theory empirically tested in the present study. Drawing on both the quantitative and qualitative findings, it is plausible that AI-supported instructional features, such as repeated low-risk practice, immediate feedback, opportunities for active participation, and visible learning progress, may have contributed to students’ higher engagement and self-efficacy. However, these potential mechanisms should be regarded as theoretically informed interpretations rather than empirically verified processes and should be directly examined in future research through explicit measurement of SDT constructs.

The theoretical implications of this paper are the extension of the self-determination theory to a multilingual university context for Chinese language learning with the support of AI. The results indicate that the AI-assisted teaching can be interpreted as a learning environment which can meet the basic psychological needs of students. Personalized practice and flexible learning experiences can foster autonomy, immediate feedback/redo of tasks can build competence, and interactive learning activities in class can build relatedness. These types of needs support explaining why students in the AI-supported group reported higher engagement and self-efficacy than those in the traditional group. The study also adds to research in technology-mediated language learning by demonstrating that engagement and self-efficacy are not independent, they are reinforcing parts of students’ learning experience. Students’ active involvement, deep processing, and visible progress helps to build their confidence in understanding and output of the Chinese language. Therefore, the study offers a theoretical explanation of the possible role of AI-assisted teaching on motivational and psychological aspects for Chinese language learning.

The results also have implications for Chinese language curriculum designers and teachers. Firstly, AI tools should be used in the classroom not to replace teachers, but to enhance pedagogy. AI can offer personalized exercises, instant feedback, pronunciation assistance, sentence-level practice, and simulated communicative exercises for the teacher. The activities can be used to involve students more actively in the process, to reflect on the use of the language and to gain confidence in using it repeatedly. Second, AI-assisted instruction should be worked out in a way that allows students to practice, tweak, and refine their content before going before their class. This is especially crucial for the student who is not feeling comfortable about incorrect pronunciation, grammar, or written expression. Last but not least, teacher guidance is crucial. Pay attention to choosing suitable AI tools, assessing the accuracy of AI feedback, aligning AI activities with course goals, and teaching students to critically apply feedback. From this perspective, successful Chinese teaching with the aid of AI requires careful teacher mediation, task design, and constant interaction in the classroom.

Building on the qualitative findings, the practical value of AI-supported instruction appears to lie not only in providing personalized learning opportunities but also in supporting specific learning processes that enhance engagement and self-efficacy. Repeated low-risk practice may reduce learners’ hesitation in using Chinese, immediate feedback may encourage deeper cognitive processing and self-reflection, and visible learning progress may strengthen learners’ confidence in both receptive and productive language use. Nevertheless, these benefits depend on appropriate teacher mediation. Teachers should carefully select AI tools, verify the accuracy and appropriateness of AI-generated feedback, align AI-supported activities with instructional objectives, and encourage students to critically evaluate AI-generated responses rather than accepting them uncritically.

### 5.4 Limitations and future directions

Some limitations of this study need to be addressed. Firstly, the sample was drawn from a single university in Malaysia, the findings of which may not be applicable to other higher education contexts or to learners from other language backgrounds. Secondly, the intervention was only 3 months long, and the long-term impact of AI-supported instruction on engagement and self-efficacy is yet to be determined. Thirdly, while the study adopted an explanatory sequential mixed methods design, the qualitative phase was conducted with a relatively few interviewees, and this may have limited the study’s ability to represent the full range of student experiences. Fourth, the study did not directly assess the underlying psychological needs in self-determination theory (autonomy, competence, and relatedness). Lastly, the intervention adopted certain AI tools and task design; variations in AI tools, platforms, or instructional approaches could yield different results. These limitations should be recognized to interpret the results prudently and to guide the development of future studies related to AI-assisted language learning.

Future research could include overcoming these challenges and continue to explore the potential of AI in language learning. Firstly, in order to improve the generalizability of research, more similar and diverse conditions in multiple universities, cultural contexts, and learner proficiency should be included. Second, longitudinal designs are warranted to explore how improvements in engagement and self-efficacy are maintained over longer time frames and how changes in students’ motivation and self-confidence occur over time across semesters. Third, future studies might include actual measures of psychological needs, autonomy, competence, relatedness, as a way to test the mechanisms posited by self-determination theory. Fourth, researchers could explore the effects of different types of AI tools, feedback modalities, and task designs to determine which features are most effective in promoting engagement and self-efficacy. Last, the use of AI-assisted instruction and teacher-guided scaffolding and peer interaction may be explored together to determine optimal ways of integrating them. Future research can focus on further investigating these aspects to deepen the understanding of how AI works and its implications in language learning environments.

## 6 Conclusion

The study used an explanatory sequential mixed-methods design to investigate the impact of AI-assisted Chinese language learning on the engagement and self-efficacy of Malaysian university students. Quantitative results revealed a significant increase in engagement and self-efficacy for the AI group compared to the traditional group, which experienced little growth. Qualitative analyses also indicated that the AI-assisted activities enabled repeated engagement, instant feedback, and repeated practice, which integrated to facilitate deeper cognitive processing, positive emotional experiences, and boost confidence in understanding and producing Chinese. These findings support the concepts of self-determination theory, where the AI-assisted learning environment met students’ psychological needs for autonomy, competence, and relatedness, encouraging them to engage in more active and self-confident learning practices. In conclusion, the study suggests that well-designed AI interventions can significantly improve the motivational and psychological aspects of language learning. The results offer valuable insights for teachers who want to use AI to support their teaching and learning, and they highlight the need for ongoing teacher interaction and task design to ensure that students are engaged and motivated to use AI effectively.

Although the present findings provide encouraging evidence for the potential of AI-supported Chinese language instruction, they should be interpreted in light of the study’s quasi-experimental design involving two intact classes from a single university. Future research should replicate the present findings across diverse educational contexts, examine the long-term effects of AI-supported instruction, and directly investigate the psychological mechanisms proposed by Self-Determination Theory through explicit measurement of learners’ basic psychological needs.

## Data Availability

The datasets presented in this study are not publicly available because they contain information that could compromise participant privacy and the conditions of ethical approval. Requests to access the datasets should be directed to the corresponding author at zhaizhaisharonwindy@gmail.com.
